# MicroRNAs and obesity-induced endothelial dysfunction: key paradigms in molecular therapy

**DOI:** 10.1186/s12933-020-01107-3

**Published:** 2020-09-09

**Authors:** Karima Ait-Aissa, Quynh My Nguyen, Mohanad Gabani, Adam Kassan, Santosh Kumar, Soo-Kyoung Choi, Alexis A. Gonzalez, Tahsin Khataei, Amal M. Sahyoun, Cheng Chen, Modar Kassan

**Affiliations:** 1 Cardiovascular Division, Department of Medicine, and Abboud Cardiovascular Research Center, University of Iowa Carver College of Medicine, Iowa City, IA 52242 USA; 2 Skaggs School of Pharmacy and Pharmaceutical Sciences, University of California, San Diego, USA; 3 Department of Pharmaceutical Sciences, School of Pharmacy, West Coast University, Los Angeles, USA; 4 Department of Physiology, College of Medicine, Brain Korea 21 PLUS Project for Medical Science, Yonsei University, Seoul, South Korea; 5 Instituto de Química, Pontificia, Universidad Católica de Valparaíso, Valparaíso, Chile; 6 Department of Food Science and Agriculture Chemistry, McGill University, Montreal, QC Canada; 7 Department of emergency and Critical Care, Shanghai General Hospital, Shanghai Jiao Tong University School of Medicine, Shanghai, China

**Keywords:** MicroRNAs, Obesity, Endothelial dysfunction, Cardiovascular diseases

## Abstract

The endothelium plays a pivotal role in maintaining vascular health. Obesity is a global epidemic that has seen dramatic increases in both adult and pediatric populations. Obesity perturbs the integrity of normal endothelium, leading to endothelial dysfunction which predisposes the patient to cardiovascular diseases. MicroRNAs (miRNAs) are short, single-stranded, non-coding RNA molecules that play important roles in a variety of cellular processes such as differentiation, proliferation, apoptosis, and stress response; their alteration contributes to the development of many pathologies including obesity. Mediators of obesity-induced endothelial dysfunction include altered endothelial nitric oxide synthase (eNOS), Sirtuin 1 (SIRT1), oxidative stress, autophagy machinery and endoplasmic reticulum (ER) stress. All of these factors have been shown to be either directly or indirectly caused by gene regulatory mechanisms of miRNAs. In this review, we aim to provide a comprehensive description of the therapeutic potential of miRNAs to treat obesity-induced endothelial dysfunction. This may lead to the identification of new targets for interventions that may prevent or delay the development of obesity-related cardiovascular disease.

## Background

Obesity is a major worldwide public health issue [1]. In the past decade, the incidence of obesity has rapidly risen to epidemic proportions [2, 3]. In the United States, obesity continues to be one of the leading public health crises. Almost a third of the American population is affected by obesity (Body mass index (BMI) > 30), and 60% fall into the overweight category (BMI > 25) [4, 5]. It is crucial to address and treat obesity because it is classified as a risk factor in the development of cardiovascular disease [6]. Diverse mechanisms by which obesity promotes cardiovascular disease have been proposed, and most involve endothelial dysfunction [7]. Vascular function assessments in subjects with obesity have demonstrated altered properties of endothelial function [8, 9]. Many studies have established that endothelial dysfunction can be considered as the first step in the progression of cardiovascular disease [10–13]. Thus, a better understanding of the mediators of obesity-induced endothelial dysfunction will help us to identify new targets for interventions that may prevent or postpone the development of obesity-related cardiovascular disease.

MicroRNAs (miRNAs) are noncoding small RNAs that play a central role in a wide range of biological cellular functions [14]. Altered microRNA expression has been reported in association with many different human diseases such as cancer, neurodevelopmental, metabolic, and cardiovascular diseases [15, 16]. Dysregulation of microRNAs affects the status and functions of different tissues and organs, including the endothelial dysfunction that leads to obesity-induced cardiovascular diseases. In this review, we will first begin by revising the biogenesis, regulation and mechanism of action of miRNAs. Then, we will illustrate the importance of miRNAs as modulators of endothelial function, and we will address the role of miRNAs in obesity-induced endothelial dysfunction.

## MicroRNAs

**Fig. 1:**
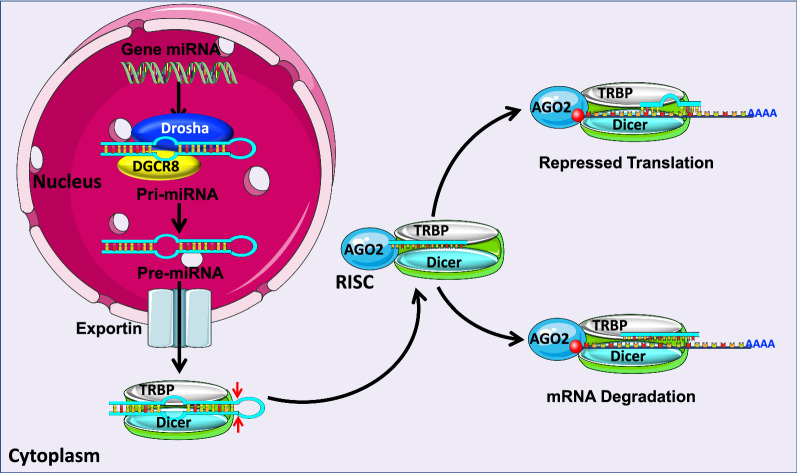
Summarized scheme of miRNAs biogenesis and mechanism of action: In the nucleus, Pol II and Pol III RNA polymerases transcribe the coding sequences of miRNAs. Drosha binds to DGCR8 cofactor to catalyze the formation of pre-miRNA. Pre-miRNA is then translocated by the exportins system from the nucleus to the cytoplasm, where it is then cleaved by the Dicer-TRBP complex to form a 22nt-dsRNA. Within the cytoplasm, the 22nt-dsRNA interacts with AGO proteins to form the RISC complex, while the passenger strand is degraded. The 22nt-RNA guide chain constitutes a mature miRNA that guides the RISC complex towards the 3-UTR regions of the mRNA targets. This interaction either represses their translation or induces their degradation. Pol: polymerase; Drosha: ribonuclease III double-stranded RNA-specific endoribonuclease; DGCR8: DiGeorge syndrome chromosomal region 8; Dicer: helicase with RNase motif; TRBP: TAR RNA binding protein; AGO: Argonaute protein; RISC: RNA-induced silencing complex; UTR: untranslated region

MiRNAs are small (18–25 nucleotides (nt)), single-stranded and non-coding RNA molecules that play important roles in multiple cellular processes such as differentiation, proliferation, apoptosis, stress response. Their alteration contributes to the development of many pathologies including obesity [17]. After the discovery of the first miRNA, lin-4, in Caenorhabditis elegans in 1993 [18], researchers have since demonstrated that these small molecules are an abundant class of RNAs in all prokaryotic and eukaryotic cells [19, 20]. To date, 2654 human miRNAs have been uncovered [21] and each single miRNA can regulate the expression of several different genes. Additionally, different miRNAs can cooperatively regulate the expression of a target gene. This fact exposes the high complexity of the regulatory network constituted by the miRNAs and their targets [22]. In mammals, miRNAs can bind, either partially or totally, to the 3′UTR regions of a wide variety of mRNAs in order to prevent their translation into proteins or induce their degradation [20] (Fig. 1).

### MiRNAs biogenesis, regulation, and mechanism of action

While most of the gene-encoding miRNAs are incorporated into the genome and are expressed using their own promoter, a minority are located within the introns and are transcribed as part of the annotated genes [20]. During miRNA biogenesis, the gene-encoding miRNA transcription is mediated mainly by RNA polymerase II (Pol II) and to a lesser extent by RNA polymerase III (Pol III), producing RNA precursors named pri-miRNAs. These pri-miRNAs are accessorized with their own CAP and poly-A tail, at 5′ and 3′ endpoints, respectively [23]. Every pri-miRNA contains a stem (~ 33 pb), a terminal loop, and a single-stranded RNA segment (ssRNA) [23, 24]. The pri-miRNAs then enter the maturation process where two ribonuclease-mediated reactions are required [20]. The process begins in the nucleus, where it is mediated by Drosha, a RNase type III protein; afterward, the process moves to the cytoplasm where it is mediated by Dicer, a type III endoribonuclease [25]. In the nucleus, Drosha binds to Di George Syndrome Critical Region Gene 8 (DGCR8) cofactor to form a complex known as a microprocessor [25, 26]. The DGCR8 part of this complex then binds to pri-miRNA’ s stem and loop structure, allowing Drosha to catalyze the breaking of the double strand of RNA in the stem. This cleavage induces the release of a fragment of 70 nt with hairpin structure called precursor miRNA (Pre-miRNA) [27, 28]. Following this, Exportin-5 and its cofactor, Ras-related nuclear Guanine nucleotide-binding proteins (Ran-GTP) export the newly formed pre-miRNAs to the cytoplasm where the second reaction of the maturation process takes place [29]. In the cytoplasm, Dicer cleaves the pre-miRNAs near the terminal loop structure, creating a double-stranded miRNA (dsRNA) fragment of approximately 22 nt [30]. Dicer additionally interacts with TAR RNA-binding protein (TRBP), contributing to the formation of the RNA-Inducing Silencing Complex (RISC) [31–33]. The newly formed 22 nt- dsRNA binds to the protein Argonaute (Ago) to generate the effector complex, RISC. The guide RNA chain of 22 nt remains in the RISC complex as a mature miRNA, while the transient chain is degraded in some cases [29]. Next, the seed sequence of the mature miRNA facilitates specific recognition of the 3-UTR regions of the target mRNAs by guiding the RISC effector complex to recognize the target mRNA and negatively signal its expression. When the complementarity of sequences between the seed sequence of the miRNA and the 3-UTR region of the target mRNA is imperfect, ribosomal access to the mRNA is blocked, thereby repressing translation. On the other hand, when the complementarity is perfect, the target mRNA is degraded [30] (Fig. 1).

## Obesity, microRNAs and endothelial dysfunction

The endothelium, a monolayer of endothelial cells (ECs) coating the vascular lumen, is the first point of contact between the blood components and the vascular wall [34–36]. Under physiological conditions, the endothelium is able to respond to physical and chemical stimuli by releasing several different factors that play key roles in regulating cellular adhesion, thromboresistance, smooth muscle cell proliferation, inflammation, and vascular tone [37, 38]. Of particular note, the endothelium responds to changes in blood flow or chemical agents by releasing molecules that induce the vessel to relax or constrict [35]. Some of these molecules, such as nitric oxide (NO), possesses vasodilatory properties [37], while others, such as endothelin [39], are vasoconstrictors. By regulating vessel tone and diameter, this vasomotion is directly involved in balancing oxygen supply with the metabolic demands of tissues [40]. The imbalance between vasoconstrictor and vasodilatory markers is a notable feature of cardiovascular diseases and will lead to vascular endothelial dysfunction [41].

Clinical studies have shown that patients with obesity display lower levels of NO bioavailability, leading to impairment of endothelium-dependent vasodilation [42]. These studies have established that decreased expression of endothelial nitric oxide synthase (eNOS), the enzyme responsible for NO production in the endothelium, is the major cause for endothelial dysfunction in obesity [8].

Furthermore, evidence indicates that the enzyme NAD-dependent deacetylase sirtuin-1 (SIRT1), which deacetylates proteins that contribute to cellular regulation during stress, plays a pivotal role in endothelial homeostasis by increasing eNOS gene expression [43]. Interestingly, SIRT1 has been found to be reduced during obesity, thereby leading to endothelial dysfunction [44].

**Fig. 2:**
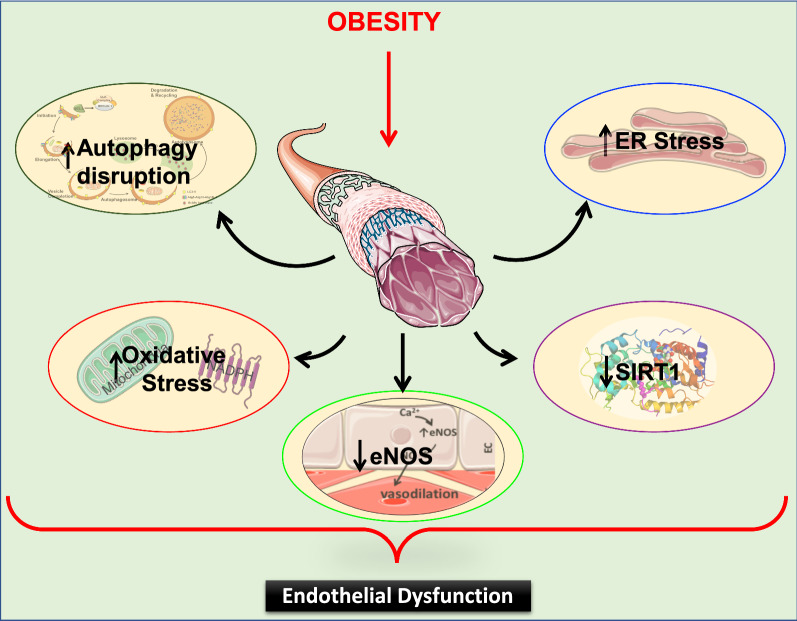
Obesity induces endothelial dysfunction. Obesity induces a decrease in NO bioavailability by: reducing eNOS activation and/or expression; negatively regulating SIRT1; and inducing cellular stresses including oxidative stress, ER stress and autophagy disruption. All of these effects lead to endothelial dysfunction. NO: Nitric Oxide; eNOS: endothelial Nitric Oxide Synthase; SIRT1: Sirtuin 1; ER: endoplasmic reticulum

Obesity has additionally been shown to induce several cellular stresses that impair endothelial function. These major stresses include oxidative stress [45], endoplasmic reticulum (ER) stress [46, 47], autophagy disruption [48], and inflammation [49]. All these stressors have been shown to decrease NO bioavailability during obesity and as a consequence, lead to endothelial dysfunction [49] (Fig. 2).

Recently, in vitro and in vivo studies have established that miRNAs are crucial for ECs gene expression, regulation and function in various pathological conditions including obesity [17, 50, 51]. In this review, we will provide an overview of the miRNA role in controlling the key connecting mechanism between endothelial dysfunction and obesity.

### Endothelial nitric oxide synthase and miRNAs

In the endothelium, NO is synthesized through the activation of eNOS [37]. Calcium (Ca^2+^) entry into the ECs activates eNOS, which then transforms l-arginine into l-citrulline, leading to NO production. NO will diffuse from the ECs to the smooth muscle cells where it activates the soluble guanylate cyclase and increases the levels of cyclic guanosine monophosphate (cGMP) thereby causing vasodilatation. A major contributing factor to decreased NO bioavailability is the downregulation of eNOS mRNA expression [37].

**Fig. 3:**
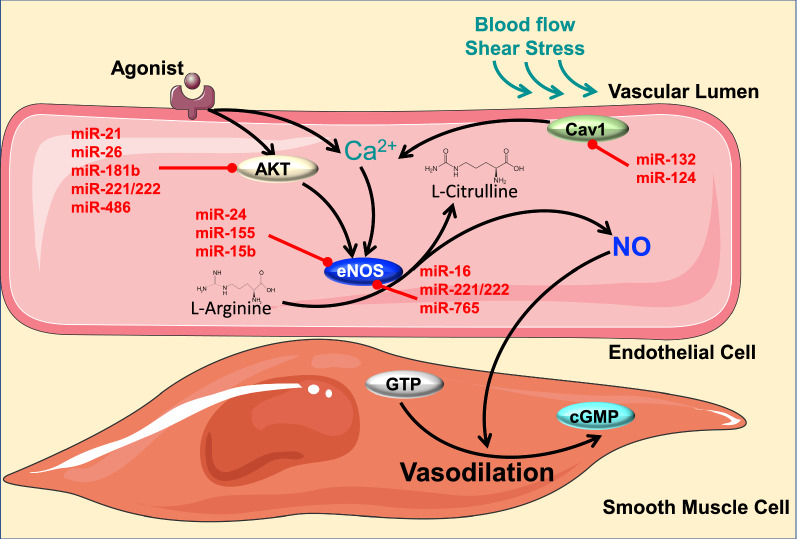
Obesity, eNOS, miRNAs and endothelial function. Ca^2+^ activates eNOS which then converts l-Arginine to l-Citrulline, producing NO as a byproduct. NO, synthetized through eNOS phosphorylation in the endothelium, will diffuse to the smooth muscle cells and increase the levels of cGMP, which then induce vasodilatation. During obesity, higher levels of miR-24, miR-155, miR-15b, miR-16, miR-221/222 and miR-765 are expressed and directly inhibit eNOS translation, thereby causing endothelial dysfunction. Other miRNAs have been shown to affect eNOS through indirect signaling pathways such as AKT (miR-21, miR-26, miR-221/222, and miR-486) and Caveolin1 (miR-132 and miR-124). eNOS: endothelial Nitric Oxide Synthase; NO: Nitric Oxide; cGMP: cyclic guanosine monophosphate; Cav1: Caveolin 1; AKT: Protein kinase B

MiRNAs have been shown to play an important role in regulating eNOS [52]. Therefore, we can consider that a loss of eNOS RNA messenger during obesity [8, 53] may be due to a change in miRNA profile during obesity. In this section, we will discuss further the interplay between miRNAs and eNOS in the context of obesity-induced endothelial dysfunction (Fig. 3).

Recent evidences have established the important role of miR-24 in regulating eNOS in the vasculature [54–57]. Interestingly, miR-24 is well known to be increased during obesity in diverse tissues including abdominal adipose tissue [58], liver [59] and hypothalamus [60]. Although there are no published studies investigating the role of miR-24 in ECs during obesity, in vitro studies have demonstrated that miR-24 significantly inhibits ECs proliferation and eNOS gene expression [54]. It is well known that endothelial dysfunction underlies the relationship between obesity and atherosclerosis [61]. eNOS deficiency accelerates atherosclerotic lesion formation in mice [62]. Similarly, miR-24 exacerbates atherogenesis and promote atheromatous plaque formation [63]. These findings suggest that further exploration of obesity-induced miR-24 upregulation may yield new therapeutic strategies for obesity-induced endothelial dysfunction.

MiR-155 has been described as an essential regulator of eNOS expression and endothelial function [64], and its inhibition is suggested to be a useful approach for restoring endothelial function during the development of cardiovascular diseases [64]. Interestingly, higher levels of miR-155 expression have been exhibited in adipose and kidney tissue from mice and patients with obesity [65, 66]. In addition, miR-155 deletion abolished high fat diet (HFD)-induced adipocyte hypertrophy and inflammation, [65] indicating a detrimental role of miR-155 in obesity. ECs pretreated with Tumor Necrosis Factor (TNF)-α, known to be increased during obesity, displayed higher levels of miR-155 [67]. Moreover, TNF-α has been shown to play an important role in endothelial dysfunction during obesity [68] and metabolic diseases [69]. All together, these studies suggest a potential therapeutic for miR-155 inhibition in obesity-induced cardiovascular diseases.

Among the miRNAs that have been shown to play a direct role in regulating eNOS are miR-15b and miR-16 [70]. Expressed in ECs, these miRNAs interact directly with eNOS by targeting its mRNA at 3UTR [70]. Interestingly, these two miRNAs are known to be increased in obesity [71, 72]. Inhibition of miR-15b reduced hepatic insulin resistance in obesity [71], while inhibition of miR-16 decreased inflammation [73]. Both insulin resistance and inflammation are frequently associated with endothelial dysfunction and have been proposed to play a major role in cardiovascular diseases [49, 74]. Although further studies are needed, these findings suggest that miR-15b and miR-16 inhibition in animal models or patients with obesity will protect against eNOS mRNA degradation and can potentially prevent obesity-induced vascular dysfunction.

Another key player in vascular endothelial biology is miR-221/222, which is known to directly target eNOS [75]. Expression of this miRNA is upregulated in ECs during atherosclerosis and obesity [75]. In particular, it has been shown that miR-221/222 downregulates eNOS expression, leading to lower NO production in the vasculature during atherosclerosis [75]. Thus, inhibiting miR-221/222 could have clinical therapeutic effect in patients with obesity.

Finally, miR-765, the most abundant miRNAs detected in ECs [76], is known to bind directly to eNOS mRNA, leading to the mRNA’s destabilization [76, 77]. Patients with coronary artery disease display high levels of circulatory miR-765, suggesting its role as a biomarker for coronary artery disease [78]. Although this study did not directly correlate miR-765 to obesity, a diversity of evidences has demonstrated that obesity is highly associated to coronary artery disease [79–81]. Therefore, the assessment of miR-765 changes in ECs during obesity would be an interesting step to consider in the pursuit of therapeutic approaches against obesity-induced endothelial dysfunction (Table 1).

**Table 1:** MiRNA regulation during obesity, their function in the cardiovascular system and their potential targets in endothelial cells (eNOS)

miRNA	Up/downregulation (obesity)	Refs.	Function	Refs.	Target	Refs.
miR-24	Up	[58–60]	Exacerbates atherogenesis and plaque formation	[62, 63]	eNOS	[54–57]
miR-155	Up	[65, 66]	Adipocytes hypertrophy and inflammation	[65]	eNOS	[64]
miR-15b	Up	[71, 72]	Hepatic insulin resistance	[71]	eNOS	[70]
miR-16	Up	[71, 72]	Increases inflammation	[73]	eNOS	[70]
miR-221/222	Up	[75]	Increases atherosclerosis	[75]	eNOS	[75]
miR-765	Unknown	–	Coronary artery disease	[78]	eNOS	[76, 77]

Other miRNAs have been shown to affect eNOS through indirect signaling pathways such as Protein kinase B (AKT) (miR-21, miR-26, miR-181b, miR-221/222, and miR-486) [82, 83] and Caveolin1 (miR-132 and miR-124) [84–86]. However, in this review, we have discussed only those miRNAs that directly affect eNOS.

### Sirtuin 1 and miRNAs

**Fig. 4:**
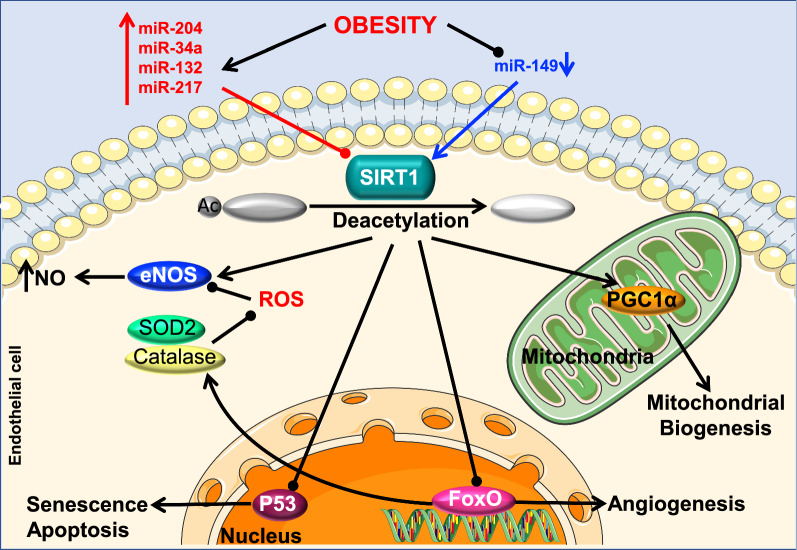
Obesity, SIRT1, miRNAs and endothelial function. SIRT1 directly deacetylates eNOS, PGC-1 α, p53, and FoxO1 leading to enhanced NO bioavailability and mitochondrial biogenesis as well as, decreased senescence, angiogenesis, apoptosis, and oxidative stress. All of these effects allow for the maintenance of endothelial homeostasis. Levels of miRNAs that directly target SIRT1 and thereby modulate endothelial function can be either increased (miR-204, miR-34a, miR-132, miR-217 and miR-200c) or decreased (miR-149) in obesity. SIRT1: Sirtuin 1; eNOS: endothelial Nitric Oxide Synthase; NO: Nitric Oxide; ROS: Reactive oxygen species; SOD2: Superoxide dismutase; P53: Tumor protein; FoxO: Forkhead box; PGC-1 α: Peroxisome proliferator-activated receptor gamma coactivator 1-alpha

Sirtuin 1 (SIRT 1) is a member of a class III histone deacetylase family proteins (HDACs) dependent on nicotinamide adenine dinucleotide (NAD^+^) [87]. SIRT1 deacetylates histone proteins in addition to transcription factors and cofactors involved in gene regulation [88]. SIRT1 is considered the major metabolic regulator because of its ability to regulate several factors involved in energy homeostasis [89, 90]. Recent evidence supports the idea that SIRT1, which is highly expressed in ECs, plays an important role in the regulation of endothelial function [91, 92]. In fact, SIRT1 is involved in regulating reactive oxygen species (ROS) production, increasing NO bioavailability, inhibiting vascular growth factors and reducing senescence modulators to maintain the endothelial homeostasis. During pathophysiological conditions, such as obesity, SIRT1 expression is altered in the vasculature leading to endothelial dysfunction [92–95]. Our recent data have demonstrated that SIRT1 is reduced in ECs from obese mice, while protecting SIRT1 preserves endothelial function by protecting caveolin-1 expression and reducing ER stress [91] (Fig. 4).

Our group has further demonstrated that miR-204 is an important factor that regulates vascular SIRT1 [91]. During obesity, miR-204 is highly expressed in ECs, leading to decreased levels of SIRT1 and endothelial dysfunction. Inhibition of miR-204 rescues impaired endothelial-dependent vasorelaxation and vascular expression of SIRT1 [91]. These findings indicate that SIRT-1 is a direct target of miR-204 and manipulating vascular miR-204 expression during obesity can potentially be a great therapeutic target against obesity-induced endothelial dysfunction.

Obesity is also known to increase endothelial senescence and as a consequence, leads to endothelial dysfunction [96, 97]. Several miRNAs have been shown to be involved in endothelial senescence, including miR-217 and miR-34a [98, 99]. These two miRNAs share a similar function as negative regulators of SIRT1 [98, 100]. Other studies have shown that overexpression of miR-217 and miR-34a in ECs from young individuals decreased SIRT1 expression and remarkably induced premature cell senescence [98, 99]. Interestingly, these two miRNAs are increased during obesity [100, 101]. From these findings, one could speculate that during obesity, these two upregulated miRNAs could influence the endothelial function through decreasing SIRT1 and inducing premature senescence pathways. Thus, further investigations of this pathway constitute a promising therapeutic strategy.

In addition, other studies have established miR-149 as an important player in preventing endothelial dysfunction [102]. MiR-149 stimulates the activity of SIRT1-induced peroxisome proliferator-activated receptor γ coactivator 1 alpha (PGC1α) and mitochondrial biogenesis [103]. Upregulation of PGC-1α can prevent the development of, and even encourage regression of, atherosclerotic lesions during obesity [104]. HFD and obesity have been shown to significantly reduce the expression of miR-149, thereby lowering SIRT1 activity [103, 105]. These findings suggest that, by restoring SIRT1, vascular miR-149 overexpression may exert protective effects on obesity-induced endothelial damage.

Similarly, miR-132 has been described as a direct regulator of SIRT1 [106]. This miRNA has been shown to be increased during obesity and to induce inflammation in ECs via reduction of SIRT1 [106–108]. Inflammation is tightly associated with vascular endothelial dysfunction in obese subjects [109]. Thus, regulating miR-132 level during obesity could have a beneficial effect on the endothelial function. All data are summarized in Table 2.

**Table 2:** MiRNA regulation during obesity, their function in the cardiovascular system and their potential targets in endothelial cells (SIRT1)

miRNA	Up/downregulation (obesity)	Refs.	Function	Refs.	Target	Refs.
miR-204	Up	[91]	EC dysfunction	[91]	SIRT1	[91]
miR-217	Up	[101]	Premature cell senescence	[98]	SIRT1	[98]
miR-34a	Up	[100]	Premature cell senescence	[99]	SIRT1	[100]
miR-149	Down	[103, 105]	Regression of atherosclerotic lesions	[104]	SIRT1	[103]
miR-132	Up	[106–108]	Macrophage infiltration in ECs	[106–108]	SIRT1	[106]

### Oxidative stress and miRNAs

**Fig. 5:**
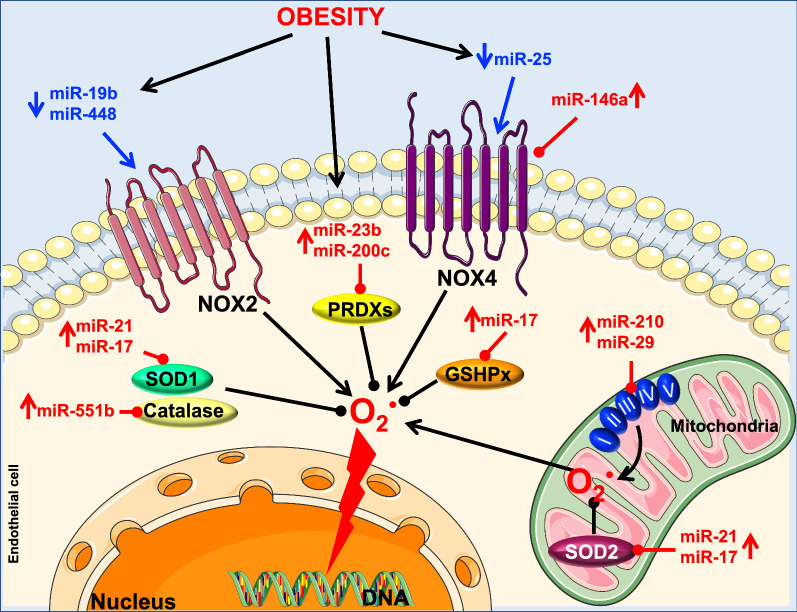
Obesity, oxidative stress, miRNAs and endothelial function. Superoxide anions (O_2_^·^) are mainly produced by NOX2, NOX4 and mitochondria through electron transport chain complex I and III. Under physiological conditions, O_2_^·^ are highly regulated by antioxidant mechanism through SOD that detoxifies O_2_^·^ to hydrogen peroxide (H_2_O_2_), which is then converted to water (H_2_O) by catalase or glutathione peroxidase (GSHPx) and peroxiredoxins (PRDXs). Obesity interferes with the normal workings of this system. By decreasing miR-448-3p and miR-19b, it increases NOX2 levels. At the same time, it decreases miR-25 while increasing miR-146a, which can cause up or downregulation of NOX4 levels respectively (NOX4 has dual effect). Additionally, obesity increases the levels of miR-210 and miR-29, both of which affect the mitochondrial respiratory chain. Moreover, obesity modulates the levels of miRNAs that regulate the antioxidant system (e.g. miR-21 and miR-17, both of which target SOD, with miR-17 additionally targeting GSHPx; and miR-23b and miR-200c, both of which target PRDXs). Altogether, the modulation of miRNA levels during obesity leads to excessive production of O_2_^·^ and consequently endothelial dysfunction. SOD: Superoxide dismutase; GSHPx: glutathione peroxidase; PRDXs: peroxiredoxins; NOX2: catalytic, membrane-bound subunit of NADPH oxidase; NOX4: NADPH oxidase homolog

Protein oxidation and ROS (free radicals) play a crucial role in maintaining normal cell physiology [110]. The mitochondria are considered as the major source of ROS in most mammalian cells [111]. More specifically, the mitochondrial respiratory chain complexes I and III, located in the inner membrane of the mitochondria, generate the majority of superoxide anions (O_2_^·^) in the cell [111–114]. Nicotinamide-adenine dinucleotide phosphate (NADPH) oxidase, a cytoplasmic enzyme, is also known to contribute to ROS production, especially in the vasculature [115]. Under physiologic conditions and because of their high reactivity with other intracellular components, ROS generation is highly regulated by antioxidant defense mechanisms that scavenge the detrimental actions of ROS [114]. The antioxidant defense systems include superoxide dismutases (SODs), glutathione peroxidase (GSHPx), catalases (CATs), and peroxiredoxins (PRDXs) [116]. For example, SODs induce the dismutation of O_2_^·^ to form H_2_O_2_ [116, 117]. At high proportions, H_2_O_2_ possesses toxic properties, but its effects are counteracted by GSHPx, CATs, and/or PRDXs [118]. Under pathophysiological conditions, an imbalance between the antioxidant system and the pro-oxidant species takes place and induces oxidative stress [119]. Studies have shown that oxidative stress by itself plays an important role in the pathogenesis of endothelial dysfunction during obesity [49]. Because of their dual roles in vascular dysfunction and obesity, the following section will discuss the potential crosstalk between ROS and miRNAs in the context of obesity-induced endothelial dysfunction (Fig. 5).

MiRNAs are potent regulators of redox homeostasis through antioxidant enzyme gene modulation [120, 121]. Mitochondrial respiratory chain complex I function depends on the usage of iron/sulfur cluster assembly enzymes (ISCU) as transient electron carriers [122, 123]. Reduction in ISCU blocks the electrons from exiting complex I, causing electron leakage and increased levels of ROS [122]. Interestingly, miR-210 is known to impair mitochondrial respiratory activity by directly targeting ISCU 1 and 2 isoforms, which then leads to superoxide anion production [124]. Additionally, recent evidence has shown that miR-210 induces ECs apoptosis during atherosclerosis [125] and is associated with cardiovascular diseases [126–129]. Although these findings have established a strong correlation between miR-210 and mitochondrial ROS production during cardiovascular diseases, further investigation is needed to show its role in obesity-induced endothelial dysfunction.

As mentioned above, miRNAs can increase cellular ROS generation/levels by directly targeting antioxidant genes [120, 129]. For instance, miR-21, expressed in ECs [52] and reported to be increased in human subjects with obesity [130, 131], directly alters SOD expression in order to promote ROS production [132, 133]. Similarly, miR-551b has been shown to enhance ROS accumulation by directly inhibiting the gene expression of either CAT [134] or FOXO3a, the transcription factor that induces SOD and CAT transcription [135]. Both SOD and CAT play a crucial role in maintaining endothelial function [136–138]. Interestingly, increased levels of miR-551b lead to endothelial dysfunction in patients with coronary artery disease [139]. Higher expression levels of miR-551b have been also reported in obesity [140]. Taken together, these findings suggest that inhibiting both miR-21 and miR-551b could prevent oxidative stress and consequently improve endothelial function in patients with obesity.

In diabetes, miR-200c is implicated in endothelial dysfunction, and its inhibition has been demonstrated to rescue endothelium-dependent vasodilation [141]. In subjects with obesity, miR-200c was found to circulate at higher levels [142, 143]. In addition, miR-200c negatively regulates redox proteins such as PRDX2, leading to accumulation of ROS [144] and consequently to senescence and apoptosis in ECs [145]. Taken all together, these findings suggest that miR-200c plays a role in the pathophysiological effects of obesity on endothelial function via oxidative stress. Additionally, miR-23b, which is expressed in ECs and plays an important role in vascular disorders [52, 146], has been shown to downregulate the antioxidant PRDX3 [147]. Furthermore, patients with cardiovascular disease risk display increased circulating levels of miR-23b [148]. These findings lead us to believe that miR-23b inhibition during obesity may induce beneficial effects on endothelial function.

Major mitochondrial antioxidant enzymes, including SOD and Gpx2, have been shown to be inhibited by miR-17 [149]. Furthermore, studies unveiled that miR-17, upregulated through inflammatory processes [150], is involved in the pathogenesis of endothelial dysfunction in the context of obesity and diabetes [151, 152]. Although miR-17 expression has been correlated to obesity-induced endothelial dysfunction, further pharmacological studies, in particular inhibitory experiments, are required to confirm its mechanisms of action.

MiR-29 isoforms have been described to affect the mitochondrial function leading to ROS production [153]. These isoforms are upregulated in animal models of metabolic disorder [154], induced endothelial cell dysfunction [155], and cellular senescence [156]. Because both endothelial dysfunction and senescence are present during obesity [97], targeting miR-29 in the vascular endothelium during obesity may have a beneficial effect on endothelial function.

NADPH oxidase (NOX) 2 and 4 are isoforms of NADPH oxidase that are highly expressed in the vasculature [115]. NOXs are known to be upregulated during obesity. This upregulation leads to excessive ROS production and consequently to endothelial dysfunction [157]. Unlike NOX4, whose activity is induced constitutively [158], the inducible NOX2 requires the intervention of p47phox and p67phox as organizer and activator, respectively [159]. NOX2-derived ROS plays a major role in models of HFD-induced endothelial dysfunction [160]. In terms of relevant miR, miR-448 plays an important role in the regulation of NOX2-derived ROS production in the heart. In fact, silencing miR-448 substantially increases NOX2-derived ROS production and facilitates the development of cardiomyopathy [161]. In patients with obesity, miR-448 is expressed at low levels [162, 163]. While there are no direct studies of this miR in ECs, we anticipate that in a model of obesity-induced endothelial dysfunction, lower levels of miR-448 will induce NOX2-derived ROS production, which will then lead to impaired endothelial function.

In vitro studies have shown that miR-19b directly inhibits p47phox-induced ROS production [164]. Moreover, miR-19b has been reported to play a key role in the attenuation of inflammation-induced EC apoptosis [165]. In addition, decreased circulating levels of miR-19b were observed in HFD-induced obese mice [166]. These findings imply that reduction of miR-19b in obesity may lead to increase in p47phox-induced ROS production and endothelial dysfunction.

NOX4-derived ROS production has been implicated in endothelial dysfunction and inflammation during obesity [167, 168]. Evidence has shown that miR-25 is expressed in ECs [169]. Interestingly, in vitro inhibition of miR-25 increases NOX4 expression and ROS levels, while its overexpression prevents NOX4-derived ROS production [170]. Rats under high cholesterol diet display lower miR-25 expression, which is associated with increased NOX4 expression levels [170]. While the relevance of these findings in the vasculature requires functional verification, this study indicates that miR-25 may directly target NOX4 to induce ROS production in ECs during obesity.

Although several papers have reported the detrimental effects of NOX4-derived ROS production in the vascular system, other studies have demonstrated the beneficial effects of NOX4 in the vessel bed. In fact, NOX4 has been reported to induce eNOS upregulation and to inhibit vascular inflammation during atherosclerosis [171, 172]. In this context, miR-146a has been shown to directly inhibit NOX4 expression, thereby causing endothelial inflammation [173]. Both animal models and patients with obesity express higher levels of miR-146a in adipose tissue [174]. Taken together, these studies point toward the importance of miR-146a in directly regulating the beneficial effects of NOX4 on the endothelium during obesity.

In summary, this section points toward the rising importance of studying the role of redox-sensitive microRNAs in order to identify more effective biomarkers and develop better therapeutic targets for the plethora of oxidative stress-related diseases, especially in the context of obesity-induced endothelial dysfunction. All data are summarized in Table 3.

**Table 3:** MiRNA regulation during obesity, their function in the cardiovascular system and their potential targets in the redox system

miRNA	Up/downregulation (obesity)	Refs.	Function	Refs.	Target	Refs.
miR-210	Unknown	–	EC apoptosis and atherosclerosis	[125]	ISCU 1,2	[124]
miR-21	Up	[130, 131]	Promotes ROS production	[132, 133]	SOD	[132, 133]
miR-551b	Up	[ [140]	Enhances ROS accumulation	[134, 135]	CAT, SOD	[134, 135]
miR-200c	Up	[142, 143]	EC apoptosis, ROS accumulation and senescence	[144, 145]	PRDX2	[144]
miR-17	Up	[151, 152]	EC dysfunction	[151, 152]	SOD, Gpx2	[149]
miR-23b	Up	[148]	EC dysfunction	[52, 146]	PRDX3	[147]
miR-29	Up	[154]	EC dysfunction, Cellular senescence	[155, 156]	ROS	[153]
miR-448	Down	[162, 163]	Attenuation of NOX2 induced-ROS accumulation	[161]	NOX2	[161]
miR-19b	Down	[166]	Attenuation of inflammation-induced EC apoptosis	[165]	p47phox	[164]
miR-25	Down	[170]	Prevent ROS production	[170]	NOX4	[170]
miR-146a	Up	[174]	EC inflammation	[173]	NOX4	[173]

### Autophagy and miRNAs

**Fig. 6:**
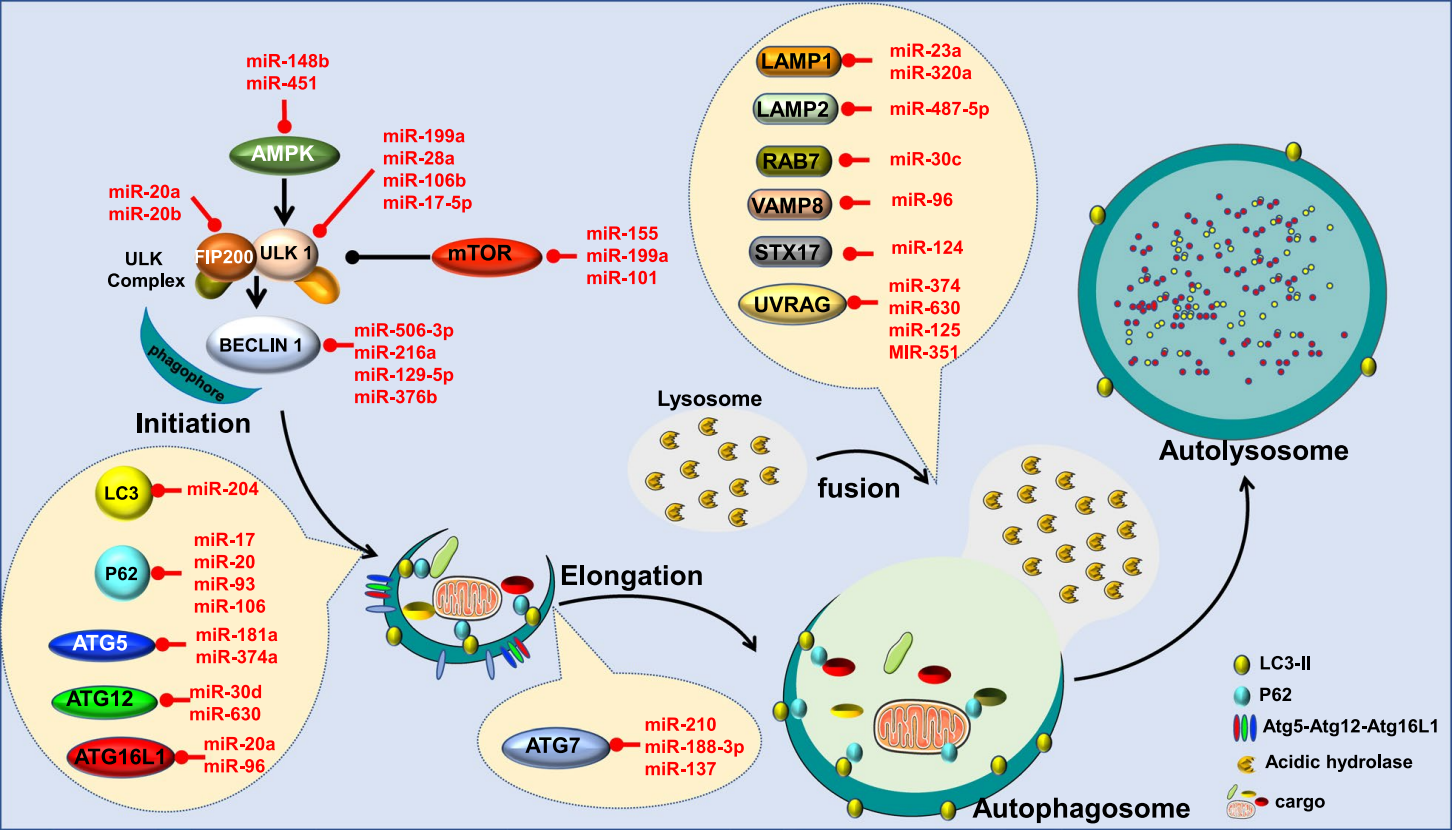
Obesity, autophagy flux, miRNAs and endothelial function. Autophagy flux consists of a series of steps including initiation, elongation, fusion of the autophagosome with the lysosome and degradation. Autophagy induction is controlled by mTOR and AMPK, which tightly regulate the ULK complex: ULK1, ATG13 and FIP200. By modulating miRNA levels, obesity regulates autophagy induction. During obesity, increased levels of miR-155, miR-199a-5b and miR-101 are expressed and all directly target mTOR. miR-148b and miR-451, also increased during obesity, target AMPK. miR-199a, miR-28a, miR-106b and miR-17-5p target ULK1, while miR-20a and miR-20b target FIP200. Beclin1, which also plays an important role in autophagy initiation, is targeted by miR-506-3p, miR-216a, miR-129-5p and miR-376b. These four miRNAs are known to be increased during obesity. Autophagosome elongation involves ATG5, ATG7, ATG 12, ATG-16L1, LC3 and p62. ATG 5 can be suppressed by miR-181a and miR-374a; ATG7 by miR-210, miR-188-3p and miR-137; ATG 12 by miR-30d and miR-630 and ATG 16-L1 by miR-20a and miR-96. All these miRNAs are increased during obesity. LC3-II is post-transcriptionally controlled by miR-204, which is known to be induced during obesity. P62 is controlled by miR-17, miR-20, miR-93 and miR-106, which are all also increased during obesity. The fusion and degradation step is controlled by UVRAG, LAMP-1/2, RAB7, VAMP8 and STX17. All of these membrane fusion factors are tightly regulated by miRNAs. UVRAG is suppressed by miR-374, miR-630, miR-125, and miR-351; Lamp1 is inhibited by miR-23a and miR-320a, while LAMP2 is inhibited by miR-487-5p; Rab7, VAMP8 and STX17 are regulated by miR-30c, miR-96, and miR-124, respectively. All of these miRNAS are induced by obesity. In summary, the modulation that takes place in miRNA levels during obesity leads to autophagy flux disruption-induced endothelial dysfunction. ULK1: unc-51-like kinase 1; FIP200: focal adhesion kinase family interacting protein of 200 kDa; mTOR: mammalian target of rapamycin; ATG: autophagy-related protein; LC3: Microtubule-associated protein 1A/1B-light chain 3; p62: sequestrome 1; UVRAG: UV resistance-associated gene; VAMP8: vesicle-associated membrane protein; STX17: Syntaxin; LAMP: Lysosome-associated membrane proteins; Rab7: Ras-related protein

Autophagy is an evolutionarily conserved mechanism that maintains cellular and nutrient homeostasis by degrading misfolded proteins and damaged organelles [175]. Dysregulation of autophagy is a major contributor to the pathogenesis of several chronic diseases including obesity-induced cardiovascular dysfunction [176, 177]. A number of recent studies have identified miRNAs that target autophagy-related proteins and that influence the autophagy flux. In this section, we will uncover the potential role of these miRNAs in the regulation of endothelial function in the context of obesity (Fig. 6).

There are three forms of autophagy: macroautophagy, microautophagy, and chaperone-mediated autophagy [175]. In this review, we will focus only on macroautophagy, which is the most important and well-studied in regard to obesity and endothelial function. Macroautophagy is characterized by three main steps: initiation and vesicle nucleation, elongation and autophagosome formation, and fusion of the autophagosome with the lysosome and degradation. The whole process is called autophagy flux. The initiation phase is orchestrated by unc-51-like kinase 1 (ULK1) complex which consists of ULK1 itself, autophagy-related protein (ATG) 13, focal adhesion kinase family interacting protein of 200 kDa (FIP200), and ATG101. The ULK1 complex drives the formation of the phagophore, the initial autophagosomal precursor membrane structure, through direct activation of vacuolar protein sorting 34 (VPS34) complex. The VPS3A complex consists of the class III phosphatidylinositol 3-kinase (PIK3C3)/VPS34, BECLIN-1, VPS15 and ATG14-like. ULK1 activity is controlled by two nutrient-regulated protein kinases, 5′ adenosine monophosphate-activated protein kinase (AMPK) and mammalian target of rapamycin (mTOR) complex 1 (mTORC1). While AMPK upregulates autophagy in response to energy depletion by directly activating ULK1, mTORC1 keeps autophagy under control through direct inactivation of ULK1.

#### MiRNAs in the regulation of autophagy initiation

As important regulators of autophagy, mTOR and AMPK are regulated by miRNAs.

miR-155, miR-199a and miR-101 directly target mTOR in different cell types [178–182]. Interestingly, all three of these miRNAs have been found at increased levels during obesity and other metabolic diseases, and have been proven to have a detrimental effect on vascular and endothelial function [65, 183–188]. The relationship between mTOR and endothelial dysfunction is well documented [189]. In fact, a study has shown that chronic inhibition of the mTOR pathway in human aortic rings leads to a reduction in endothelium-mediated vasodilation [190]. Moreover, obesity has been proven to reduce the activity of the mTOR pathway and lead to endothelial dysfunction in vasculature of animal models of obesity [191]. The reactivation of the mTOR pathway in these models improves microvascular endothelium-dependent vasodilation [191]. Altogether, these data provide a solid link between the miRNAs, obesity and endothelial dysfunction through mTOR regulation.

AMPK is also targeted by miRNAs, including miR-148b and miR-451 [192, 193]. miR-148b is stimulated during obesity [194] and plays an important role in hyperglycemia-induced endothelial dysfunction [155]. miR-451 overexpression induces AMPK inhibition, which then leads to mTORC1 over-activation [195]. In addition, microarray analysis of the heart tissue of mice on HFD has shown that, by reducing AMPK, miR-451 plays a role in the development of cardiac hypertrophy [193]. While this study uncovered the link between miR-451 and AMPK during obesity, miR-451′s role in the vascular endothelium and its contribution to the endothelial dysfunction in the context of obesity remains to be examined. The link between AMPK and endothelial dysfunction has been studied in HFD-induced animal models of obesity [196]. In this study conducted by Ma et al., HFD led to aortic AMPK downregulation and endothelial dysfunction [196]. Other studies have also shown that pharmacological activation of AMPK leads to markedly improved endothelial function [197]. Therefore, manipulating miRNAs that target AMPK during obesity could be a relevant therapeutic tool to restore endothelial function.

A recent study has uncovered the protective role of ULK1 in obesity-induced cardiac damage [198]. Obesity decreases ULK1 and leads to lipotoxicity in cardiomyocytes and consequently heart dysfunction [198]. Despite the preventive role of ULK1 in the context of obesity-induced damage, the relation between ULK1 and endothelial dysfunction in obesity has not been studied. However, there is evidence that miR-199a inhibits ULK1 complex, which then causes vascular endothelial impairment and consequently hypertension [185]. Interestingly, miR-199a is increased during obesity [182, 188], suggesting that manipulating this miRNA could restore endothelial function during obesity. Other studies have focused on miR-20a, miR-106b and miR-17-5p, three miRNAs proven to affect ULK1 complex [199–202]. Compared to healthy individuals, subjects with obesity display increased levels of miR-20a [143] which has been shown to regulate EC migration and angiogenesis, two important processes for endothelial function [203]. Similarly, miR-106b has been found to be highly expressed during metabolic disorders, in which it induces mitochondrial dysfunction [204]; its knockdown counterpart decreases endothelial proliferation and prevents atherosclerosis [205]. Likewise, miR-17-5p expression differs significantly between patients with and without obesity [206]. Downregulation of this miRNA improves cardiac function after a myocardial infarction through attenuation of EC apoptosis [207]. Altogether, these studies point toward the importance of the ULK1 in the cardiovascular system and more specifically endothelial function. Therefore, monitoring the miRNAs that regulate the ULK1 complex during obesity would have positive effects on the vascular bed.

Among the other players of autophagy initiation, miR-20a and miR-20b have been shown to negatively regulate autophagy via targeting FIP200 [208]. Both miR-20a and miR-20b have been found at higher levels in patients with obesity [143, 209] and have been demonstrated to promote senescence in human microvascular ECs [210, 211]. Regulating these miRNAs, which regulate FIP200 during obesity, could have a beneficial effect on the endothelial function.

The reduction of Beclin 1 is associated with vascular ECs injury in hypertensive patients. In this study, Zhong et al. showed that downregulating Beclin 1 expression in hypertensive patients via miR-506-3p upregulation, aggravates injury in vascular ECs [212]. Although the association between hypertension and obesity has been extensively studied and established [213–215], no study has linked miR-506-3p to obesity. Establishing this connection could constitute an important advancement in obesity-induced vascular dysfunction therapy. Another player in the regulation of autophagy initiation is miR-216a. Mir-216a controls ox-LDL induced autophagy in ECs by regulating intracellular levels of Beclin1 and may have a therapeutic role in the pathogenesis of cardiovascular disorders and atherosclerosis [216]. MiR-129-5p, a known tumor suppressor [217], is induced during HFD and alters endothelial autophagy by directly binding to Beclin 1 [218]. Similarly, miR-376b, which is also found at high levels in subjects with obesity [219], targets Beclin 1 and induce autophagy impairment in different cell types [220]. Interestingly, a study has uncovered the role of miR-376b in metabolic disease-induced EC dysfunction [155], suggesting that targeting this miRNA is a promising approach to regulate autophagy and endothelial function during obesity. While the previous studies help establish a strong relationship between miRNAs and Beclin 1 in obesity, their relationship to obesity-induced endothelial dysfunction requires further exploration. All data are summarized in Table 4.

**Table 4:** MiRNA regulation during obesity, their function in the cardiovascular system and their potential targets in autophagy induction machinery

miRNA	Up/downregulation (Obesity)	Refs.	Function	Refs.	Target	Refs.
miR-155	Up	[65]	EC dysfunction	[183]	mTOR	[178, 181]
miR-199a	Up	[184, 188]	EC dysfunction	[185]	mTOR, ULK1	[178, 180, 182, 185]
miR-101	Up	[186, 188]	EC dysfunction	[187]	mTOR	[178, 179]
miR-451	Up	[193]	Cardiac hypertrophy	[193]	AMPK	[193, 195]
miR-148b	Up	[194]	Hyperglycemia-induced EC dysfunction	[155]	AMPK	[192]
miR-20a	Up	[143]	Regulates EC migration, angiogenesis and senescence	[203, 211]	ULK1, FIP200	[199, 208]
miR-20b	Up	[209]	Promotes EC senescence	[210, 211]	FIP200	[208]
miR-106b	Up	[204]	EC proliferation and atherosclerosis	[205]	ULK1	[199, 201]
miR-17-5p	Up	[206]	ECs apoptosis and cardiac dysfunction	[207]	ULK1	[202]
miR-506-3p	Unknown	–	Aggravates injury in vascular EC	[212]	Beclin 1	[ [212]
miR-216a	Unknown	–	Controls ox-LDL induced autophagy	[216]	Beclin 1	[216]
miR-129-5p	Up	[218]	Alters EC autophagy	[218]	Beclin 1	[218]
miR-376b	Up	[219]	Autophagy impairment, EC dysfunction	[155, 220]	Beclin 1	[220]

#### MiRNAs in the regulation of autophagy elongation

After vesicle nucleation, elongation of the phagophore’s membrane constitutes a fundamental step in the formation of a complete and functional autophagosome. This process involves ATG proteins (i.e. ATG 3, 4, 5, 7, 8, 10, 12, and 16L) and the microtubule-associated protein 1 light chain (LC3) [221]. The ATG system is required for the LC3 to conjugate to phosphotidylethanolamine (PE) phospholipids and to form LC3 II. LC3 II is important for the expansion of the membrane, where it plays a crucial role in cargo recognition via its interaction to sequestrome 1, also known as the ubiquitin-binding protein p62 [221].

Similar to the components of autophagy initiation, ATG proteins are regulated by multiple miRNAs. Beyond its major role in the autophagy elongation, ATG5 holds a pivotal part in the process cell survival [222]. As such, a variety of different miRNAs take part in its regulation. For instance, miR-181a is an active inhibitor of ATG5 [223]. miR-181a is upregulated in human atherosclerosis plaques and involved in oxidative stress-induced EC dysfunction through by directly targeting autophagy machinery [224]. Moreover, it is found to be associated with the pathogenesis of obesity, [225] and its downregulation has the potential to reverse these effects [226]. In a similar manner, miR-374a has been suggested to regulate ATG5 [227]. MiR-374a has been linked to metabolic disorder during obesity [228] and can promote heart dysfunction by negatively regulating vascular endothelial growth factor (VEGF) receptor-1 signaling [229].

MiR-30d, shown to directly target and suppress ATG12 [230], is found at increased levels in subjects with obesity [58]. Although its implication in vascular function has not been assessed yet, existing data leads us to assume that downregulation of miR-30d in models with obesity, will induce a beneficial effect on endothelial function. Likewise, miR-630 suppresses autophagy by targeting ATG12 [230]. Interestingly, miRNA-630 has been designated as a putative key mediator of vascular function and cardiovascular disease risk in patients with obesity [231].

Similar to ATG12, ATG7 is also implicated in the development of atherosclerosis [232]. This protein is shown to be negatively targeted by miR-210, miR-188-3p and miR-137 [233–235]. MiR-210 is involved in ECs apoptosis during atherosclerosis [125] and is associated with cardiovascular diseases [126–129]. Comparably, miR-188-3p upregulation results in the induction of atherosclerosis [236], a disease that has been widely associated with obesity [61]. In regard to miR-137, which is also involved in obesity [237], studies have established that high glucose levels induce dysfunction of human ECs by upregulating miR-137 [238]. This suggests the involvement of miR-137 in the pathogenesis of obesity-induced endothelial dysfunction.

ATG16L1 has been shown to be another target of miRNAs such as miR-20a [239] and miR-96 [240]. Interestingly, both of these miRNAs are found to be upregulated during obesity [17, 241], and studies have demonstrated their implication in the functionality of the vascular system [203, 242]. This indicates their potential involvement in the pathogenesis of obesity-induced vascular damage.

As important markers of autophagy process, LC3 and p62 are also regulated by miRNAs. To be specific, LC3 is targeted by miR-204 [243]. Our group has previously shown that obesity is associated with increased levels of vascular miR-204, which lead to endothelial dysfunction [44]. From our findings, we believe that further exploring the mechanisms underlying obesity-induced endothelial damage with respect to miR-204 and autophagy could represent a novel strategy for obesity treatment and prevention. On the other hand, p62 has been shown to be targeted by the miR-17/20/93/106 family of miRNAs [244]. Most of these family members are involved in endothelial dysfunction. In fact, when induced by VEGF, miR-17 triggers ECs to switch to an angiogenic phenotype which has been linked to atherosclerosis and obesity [245]. As discussed above, miR-20a, miR-20b and miR-106 are all involved in the pathogenesis of obesity and endothelial dysfunction [17, 143, 192, 203, 205, 209–211]. Finally, miR-93, which plays an important role in adipogenesis [246], has been shown to regulate endothelial glycolysis and proliferation [247, 248] and to contribute to coronary atherosclerosis pathogenesis [239]. All data are summarized in Table 5.

**Table 5:** MiRNA regulation during obesity, their function in the cardiovascular system and their potential targets in autophagy elongation and nucleation machinery

miRNA	Up/downregulation (Obesity)	Refs.	Function	Refs.	Target	Refs.
miR-181a	Up	[225, 226]	Oxidative stress-induced EC dysfunction and atherosclerosis	[224]	ATG5	[223]
miR-374a	Up	[228]	Negatively regulates vascular endothelial growth factor receptor-1 signaling	[229]	ATG5	[227]
miR-30d	Up	[58]	Unknown	–	ATG12	[230]
miR-630	Up	[231]	Suppresses autophagy	[231]	ATG12	[230]
miR-210	Up	[125]	EC apoptosis and atherosclerosis	[125]	ATG7	[233]
miR-188-3p	Unknown	–	Induction of atherosclerosis	[236]	ATG7	[234]
miR-137	Up	[237]	EC dysfunction	[238]	ATG7	[235]
miR-20a	Up	[17]	EC dysfunction	[203]	ATG16L1	[239]
miR-96	Up	[241]	EC dysfunction	[242]	ATG16L1	[240]
miR-204	Up	[44]	EC dysfunction	[ [44]	LC3	[243]
miR-17	Up	[206]	Triggers a switch of EC to an angiogenic phenotype	[245]	P62	[244]
miR-93	Up	[246]	Regulates EC glycolysis and proliferation	[239, 247, 248]	P62	[244]
miR-20a	Up	[17]	Regulates EC migration, angiogenesis and senescence	[203]	P62	[244]
miR-20b	Up	[209]	Promotes EC senescence	[210]	P62	[244]
miR-106	Up	[204]	EC proliferation and atherosclerosis	[205]	P62	[244]

#### MiRNAs in autophagy fusion and degradation regulation

Once the autophagosome has formed and matured, it fuses with a lysosome to form an autolysosome. The inner membrane of the former autophagosome and the engulfed cargo are then degraded by acid hydrolases [249]. Autophagosome–lysosome fusion machinery includes UV resistance-associated gene (UVRAG), Soluble *N*-ethylmaleimide sensitive factor (NSF) Attachment Protein Receptor (SNARE) complexes, vesicle-associated membrane protein (VAMP) 8, Syntaxin (STX) 17, Lysosome-associated membrane proteins (LAMP) 1/2 and RAB 7 [250]. Although poorly studied, a number of miRNAs have been reported to regulate the fusion of autophagosome and lysosome [251]. Studies have established that lysosomal dysfunction occurs and is involved in the pathology of obesity [252]. In addition, lysosomal dysfunction has been proven to induce ECs apoptosis and senescence [253]. Therefore, regulating the late phase of autophagy during obesity could bring new insight into therapeutic approaches for vascular diseases.

UVRAG, an important player in the fusion process, is targeted by miR-374, miR-630, miR-125 and miR-351 [227, 254]. The characteristics of both miR-374 and miR-630 were discussed in the previous sections. miR-125 is implicated in the process of atherosclerosis development [255] and ECs senescence [256]. miR-351 is upregulated in the serum and ECs of atherosclerotic animal models and has been shown to attenuate ECs survival [257].

Lamp1, a primary marker of the lysosome fusion process, is a target of both miR-23a [258] and miR-320a [259]. MiR-23a plays an important role in the development of atherosclerotic plaques [260] and is a potential marker for coronary artery disease [261]. miR-320a, on the other hand, has altered circulating levels in metabolic diseases [262] and is considered a key regulator of atherogenesis [263].

Lamp2 is suppressed by miR-487-5p, [264] which has been found at higher levels in subjects with obesity [140]. Although no direct evidence has shown the implication of this miRNA in the vasculature, because Lamp2 is so crucially involved vascular function and integrity [265], we believe that we may gain some important insights by exploring this mechanism in the context of obesity-induced endothelial dysfunction.

Implicated in atherosclerosis [266], miR-30c is an efficient positive regulator of Rab7 [267]. Interestingly, both HFD and obesity downregulate miR-30c expression [268, 269] exacerbating the inflammatory process and cardiomyopathy. Although no evidence indicates that these processes would take place in the vascular bed in the context of obesity, data have demonstrated that restoration of autophagy flux through Rab7 in ECs reverses endothelial damages [270].

Finally, along the same lines, miR-96 has been predicted to bind to VAMP8 [271], and miR-124 to STX17 [272]. Both miR-96 and miR-124 have been linked to metabolic disorders and EC impairment [241, 273–275].

In the above section, we have listed the miRNAs and dissected their regulatory roles in the autophagy flux pathway; however, the interplay between autophagy machinery and miRNAs is much more complex. DICER and the AGO2, enzymes that regulate miRNAs biogenesis [276], are direct targets of the autophagolysosomal degradation. This indicates that autophagy and miRNA interactions work both ways and controlling one way would lead to a disruption of other processes and alter other physiological cellular functions. Thus, it is important to consider every single pathway that each miRNA is involved in, before exerting any potential manipulations in the context of obesity-induced vascular events. All data are summarized in Table 6.

**Table 6:** MiRNA regulation during obesity, their function in the cardiovascular system and their potential targets in autophagy fusion and degradation machinery

miRNAs	Up/downregulation (Obesity)	Refs.	Function	Refs.	Target	Refs.
miR-374	Up	[228]	Deregulating vascular endothelial growth factor receptor-1 signaling	[229]	UVRAG	[227]
miR-630	Up	[231]	Suppresses autophagy	[231]	UVRAG	[227]
miR-125	Up	[255, 256]	EC senescence and atherosclerosis	[255, 256]	UVRAG	[254]
miR-351	Up	[257]	Attenuate EC survival	[257]	UVRAG	[254]
miR-23a	Up	[261]	Promotes atherosclerotic plaque formation	[260]	Lamp1	[258]
miR-320a	Up	[262]	Regulates atherogenesis	[263]	Lamp1	[259]
miR-487-5p	Up	[140]	Unknown	–	Lamp2	[264]
miR30c	Down	[268, 269]	EC inflammation	[268, 269]	Rab7	[267]
miR-96	Up	[241]	EC impairment	[274]	VAMP8	[271]
miR-124	Up	[273]	EC apoptosis	[275]	STX17	[272]

### Endoplasmic reticulum (ER) stress and miRNAs

**Fig. 7:**
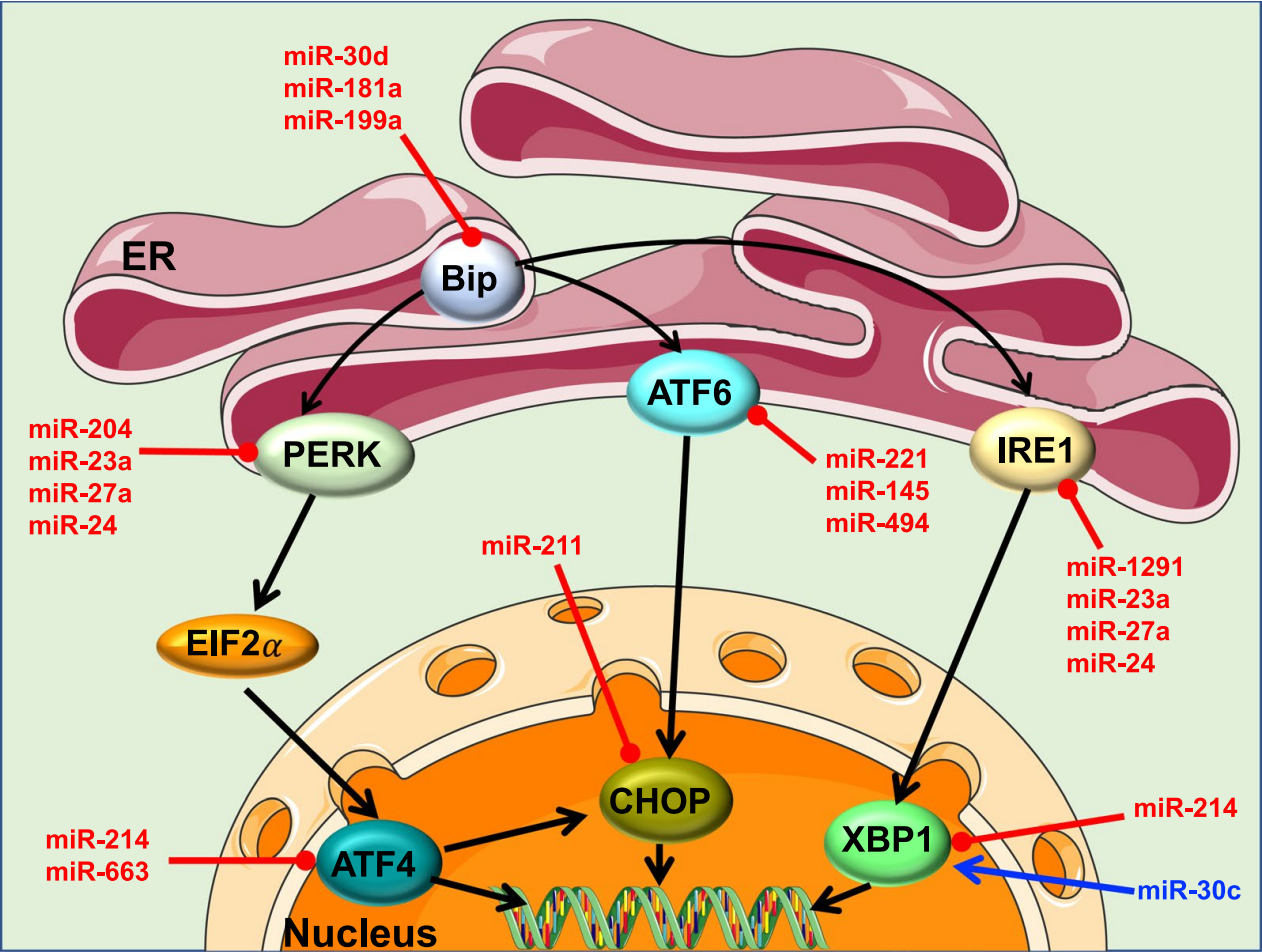
Obesity, ER stress, miRNAs and endothelial function. Under ER stress, BIP will activate the three UPR sensor pathways that are initiated by PERK, ATF6, and IRE1. MicroRNAs involved in the UPR pathway and evidenced linkage to obesity and endothelial dysfunction are listed in this diagram. miR-30d, miR-181a, and miR-199a-5p have all been shown to directly target BIP. MiR-204, miR-23a, miR-27a and miR-24 directly target and inhibit PERK signaling, which then causes ER-stress-induced cell death. ATF4, located downstream of PERK, is directly inhibited by miR-214 and miR-663. CHOP, which is regulated by ATF4, is suppressed by miR-211. IRE1α and its downstream signaling XBP1 make up the second arm of the UPR; the former is targeted by miR-1291, miR-23a, miR-27a and miR-24, while the latter is targeted by miR-214. Finally, in the third arm of UPR, miR-221, miR-145 and miR-494 directly target ATF6. ER: endoplasmic reticulum; ATF6: Activating transcription factor 6; IRE1α inositol requiring enzyme 1 alpha; and PERK protein kinase-like ER kinase; BIP: Binding Immunoglobulin Protein, also known as GRP78; UPR: unfolded protein response, ATF 4: Activating transcription factor 4; CHOP: CCAAT-enhancer-binding protein homologous protein; XBP1: X box binding protein 1; EI2 α: Eukaryotic Initiation Factor 2 alpha

The endoplasmic reticulum (ER) is the most important subcellular membrane organelle. It plays a crucial role in protein synthesis and folding, as well as in the control of cellular calcium concentrations [277]. If the protein synthesis and folding process fails, proteins will accumulate inside the ER, automatically triggering a defense mechanism called unfolded protein response (UPR). UPR represses protein synthesis and increases ER chaperone content in a bid to restore normal ER function [278]. In mammalian cells, the UPR is activated by three specialized arms: protein kinase R-like ER kinase (PERK), inositol-requiring enzyme (IRE) 1, and activating transcription factor (ATF) 6. Under normal conditions, 78 kDa-glucose-regulated protein/immunoglobulin binding protein (GRP78/BIP), an ER chaperone, binds to and maintains the inactivity of IRE1α, PERK, and ATF-6. Under stressful conditions such as misfolded protein accumulation (also known as ER stress), GRP78/BIP dissociates from IRE1α, PERK, and ATF-6, activating them and l restructuring the UPR mechanism. Once activated, UPR alleviates protein load by halting transcription, slowing translation, and targeting misfolded proteins for degradation [279]. However, chronic ER stress can deleteriously affect cells and tissues by activating the inflammatory and apoptotic pathways [280]. ER stress has been implicated in the pathogenesis of several obesity-related disorders including diabetes, atherosclerosis, and endothelial dysfunction [281–283]. Recent studies have linked microRNAs to the UPR pathway [284]. In this section, we will dissect the different pathways that link miRNAs and ER stress in regard to obesity-induced endothelial dysfunction (Fig. 7).

GRP78/BIP, a major ER chaperone protein, is critical for protein quality control and has been described as a master regulator of ER stress because of its anti-apoptotic properties [285]. Recent evidence has indicated GRP78/BIP as a novel regulator of vascular endothelial dysfunction [286, 287]. Several miRNAs, including miR-30d, miR-181a, and miR-199a, directly target GRP78/BIP [288]. In several sections prior, we have discussed the potential involvement of each of these miRNAs in the pathogenesis of obesity-induced endothelial dysfunction [58, 182, 188, 224, 225].

MiR-204 affects ER-stress in human cells [289] by directly targeting and inhibiting PERK signaling and thereby causing ER-stress-induced cell death [290]. Our group has previously demonstrated that an increase in miR-204 expression in ECs during obesity is associated with increased ER stress, while inhibition of miR-204 downregulates ER stress and restores endothelial function [91].

The transcriptional factor ATF 4, located downstream of PERK, has been implicated in microvascular endothelial regulation pathways [291] and is suggested to have a role in the pathologic angiogenesis in atherosclerotic plaques [292]. This transcription factor is targeted and inhibited by miR-214 [293]. MiR-214 regulates endothelial function and is altered in obesity [294, 295]. Another miR that is notable for its effects on ATF 4 is miR-663. Inhibition of miR-663 in aortic and venous ECs reduces ATF 4 levels [296], indicating that this miRNA may regulate expression of ATF 4. This microRNA has been linked to atherosclerosis [297] and demonstrated to regulate flow conditions, such as oscillatory shear in ECs, which is believed to contribute to atherosclerosis [298].

CCAAT-enhancer-binding protein homologous protein (CHOP), a proapoptotic transcription factor that is involved in the downstream cascade of ATF 4 [299], is a target of miR-211 [300]. While the circulatory levels of miR-211 are found to be increased in patients with atherosclerosis [301], no evidence has linked miR-211 to endothelial function, despite the extensive number of studies establishing the interconnection between CHOP, ER stress, atherosclerosis and endothelial dysfunction [302]. We believe that further exploring the role of miR-211 in the pathogenesis of obesity-induced endothelial dysfunction through ER stress pathways will uncover new mechanisms and offer a better understanding of the pathology.

A part of the UPR, IRE1α is regulated by miR-1291 [303]. Although little is known about its involvement in the obesity-induced vascular damage pathogenesis, miR-1291 has been shown to be increased in patients with myocardial infarction [304], a disease whose incidence is augmented by obesity [305] and is linked to endothelial dysfunction [306].

In the downstream signaling cascade, X-box binding protein 1 (XBP1) is a target of miR-214 and miR-30c [307, 308]. The implication of both miR-214 and miR-30c in the process of obesity-induced endothelial damage has been discussed earlier in this review.

Induction of the miR-23a-27a-24 cluster activates PERK and IRE1, which then leads to perturbation in intracellular calcium levels and mitochondrial function [309]. As mentioned above, miR-23a and miR-24 are associated to the pathogenesis of obesity and vascular endothelial damage [54, 55, 58, 63, 260, 261]. miR-27a is upregulated during obesity [310] and involved in atherosclerosis [311].

As the third arm of the UPR, ATF6 is not spared from miRNA regulation. MiR-221, miR-145 and miR-494 are shown to be strong candidates in this matter [312]. Interestingly, miR-221 is elevated during obesity [313] and associated to EC dysfunction in the progression of atherosclerosis [314]. Both miR-145 and miR-494 are related to obesity [315, 316] and atherosclerosis [317–319].

UPR by itself can exert a regulatory role on miRNAs; this aspect has been well described in the literature but not discussed here [284, 320]. Throughout this section, we have dissected the potential role that miRNAs may play on ER stress to exacerbate obesity-induced vascular damage. With these findings in mind, we believe that manipulation of each miRNA could offer a novel mechanism and therapeutic approach to prevent endothelial dysfunction during obesity. All data are summarized in Table 7.

**Table 7:** MiRNA regulation during obesity, their function in the cardiovascular system and their potential targets in ER stress pathway

miRNAs	Up/downregulation (Obesity)	Refs.	Function	Refs.	Target	Refs.
miR-30d	Up	[58]	Unknown	–	GRP78/BIP	[288]
miR-181a	Up	[225, 226]	Oxidative stress-induced EC dysfunction	[224]	GRP78/BIP	[288]
miR-199a	Up	[184, 188]	EC dysfunction	[185]	GRP78/BIP	[288]
miR-204	Up	[44]	EC dysfunction	[44]	PERK	[289, 290]
miR-214	Up	[294, 295]	Regulates EC function	[294, 295]	ATF 4, XBP1	[293, 307]
miR-663	Up	[297]	Regulates oscillatory shear in EC- atherosclerosis	[297]	ATF 4	[296]
miR-211	Up	[301]	Promotes atherosclerosis	[302]	CHOP	[300]
miR-1291	Unknown	–	EC dysfunction	[305, 306]	UPR, IRE1α	[303]
miR30c	Down	[268, 269]	EC inflammation	[268, 269]	XBP1	[308]
miR-24	Up	[58–60]	Exacerbates atherogenesis and plaque formation	[62, 63]	PERK&IRE1	[309]
miR-23a	Up	[261]	Promotes atherosclerotic plaque formation	[260]	PERK&IRE1	[309]
miR-27a	Up	[310]	Atherosclerosis formation	[311]	PERK&IRE1	[309]
miR-221	Up	[313]	EC dysfunction	[314]	ATF6	[312]
miR-145	Up	[315]	Promotes atherosclerosis	[317]	ATF6	[312]
miR-494	Up	[316]	Promotes atherosclerosis	[318, 319]	ATF6	[312]

## Challenges associated with clinical application

The miRNA research field has extensively grown in the last decade; however, the development of miRNA-based therapy remains very challenging. MiRNAs manipulation mainly uses gain- and loss-of-function tools such as miRNA mimics, and antagonists and inhibitors, respectively. The miRNA mimic technology is an innovative approach for overexpressing the mature sequence of the miRNA. Although miRNA mimics are advantageous in that they are easily synthesized and to behave in the same manner as their analog endogenous miRNA, they can lead to undesirable off-target effects because they non-selectively imitate the endogenous miRNA of even normal cells or tissues. The miRNAs antagonists, known as antagomirs, are 22–23nt RNA analogs that have a complementary sequence to the miRNA and silence the miRNA’s function. Despite the advantages of nuclease resistance and their easy-delivery system, antagomirs often have off-target effects and induce unexpected side-effects. This occurs mostly because each miRNA can have different targets depending on the cell or tissue type or because other miRNAs are acting on the same mRNA. Few studies have attempted to introduce these tools into clinical practice. One such study involves MRX34, a liposomal miR-34a mimic that is one of the first miRNA mimic to be put to clinical application; in 2013, it was used as miRNA cancer therapy in patients with liver cancer and hematological malignancies [321]. Due to the adverse effects of this miRNA mimic, the study had to be discontinued before it reached phase II of the clinical trial. MRG-201, which mimics miR-29 and was being studied to determine its ability to limit the formation of fibrous scar tissue, has seen more success up until this point [322]. It has successfully passed phase I of the clinical trial, and a phase II clinical trial is soon-to-be initiated, with the purpose of evaluating its efficacy in subjects with fibrotic diseases such as keloids formation.

Within the antagonist and inhibitor category, Miravirsen, a miR-122 inhibitor, has been established to effectively combat hepatitis C viral infection [323]. Miravirsen is currently in phase II of the clinical trial and has shown beneficial outcomes without any notable adverse effects. With its ability to downregulate miR-122, this drug is currently a very promising combatant against hepatitis C. Likewise, MRG-106, a synthetic inhibitor of miR-155 that plays an important role in cutaneous T cell lymphomas, has shown very promising results from phase I of its clinical trial. This has encouraged the investigation of MRG-106 to be continued and to include patients with additional hematological malignancies in which miR-155 is known to be elevated and relevant.

## Conclusion and future perspectives

The evidence that we have presented here in this review, helps to strengthen the concept that miRNAs constitute a good target for a based therapy tool against obesity-induced endothelial damage. However, their multitude of targets and their interplay with different organs and subcellular organelles, makes them very non-specific and therefore prone to unwanted effects, should they be manipulated. Therefore, extensive bench side investigations are essential to harness the therapeutic potential of miRNAs while minimizing undesirable off-target effects. Investigations is most needed at the bench level. The achievement of this goal would constitute a breakthrough in current medicine.

## Data Availability

Not applicable.

## Abbreviations

miRNAs: microRNAs
Pol: polymerase
Drosha: ribonuclease III double-stranded RNA-specific endoribonuclease
DGCR8: DiGeorge syndrome chromosomal region 8
Dicer: helicase with RNase motif
TRBP: TAR RNA binding protein
AGO: argonaute protein
RISC: RNA-induced silencing complex
UTR: untranslated region
eNOS: endothelial nitric oxide synthase
SIRT1: NAD-dependent deacetylase sirtuin-1
ER: endoplasmic reticulum
EC: endothelial cells
Cav1: caveolin 1
cGMP: cyclic guanosine monophosphate
TNF-α: Tumor Necrosis Factor
ROS: reactive oxygen species
PGC1α: peroxisome proliferator-activated receptor γ coactivator 1 alpha
O_2_^•^: superoxide anion
NADPH: nicotinamide-adenine dinucleotide phosphate
NOX: catalytic, membrane-bound subunit of NADPH oxidase
SODs: superoxide dismutases
GSHPx: glutathione peroxidase
CATs: catalases
PRDXs: peroxiredoxins
ISCU: iron/sulfur cluster assembly enzymes
ULK1: UNC-51-like kinase 1
FIP200: focal adhesion kinase family interacting protein of 200 kDa
VPS34: vacuolar protein sorting 34
PIK3C3: class III phosphatidylinositol 3-kinase
AMPK: 5′ adenosine monophosphate-activated protein kinase
mTORC1: mammalian target of rapamycin (mTOR) complex 1
ATG: autophagy-related protein
LC3: microtubule-associated protein 1A/1B-light chain 3
P62: sequestrome 1
FoxO: forkhead box
PGC-1 α: peroxisome proliferator-activated receptor gamma coactivator 1-alpha
UVRAG: UV resistance-associated gene
VAMP8: vesicle-associated membrane protein
STX17: syntaxin
LAMP: lysosome-associated membrane proteins
Rab7: ras-related protein
PE: phosphotidyl-ethanolamine
VEGF: vascular endothelial growth factor
NSF: soluble *N*-ethylmaleimide sensitive factor
SNARE: Attachment Protein Receptor
UPR: unfolded protein response
PERK: protein kinase R-like ER kinase
IRE 1: inositol-requiring enzyme
ATF 6: activating transcription factor
GRP78/BIP: 78 kDa-glucose-regulated protein/immunoglobulin binding protein
ATF 4: activating transcription factor 4
CHOP: CCAAT-enhancer-binding protein homologous protein
XBP1: X box binding protein 1
EI2 α: Eukaryotic Initiation Factor 2 alpha
